# Examining the influence of correlates on different quantile survival times: infant mortality in Bangladesh

**DOI:** 10.1186/s12889-022-14396-y

**Published:** 2022-10-28

**Authors:** Ahsan Rahman Jamee, Kanchan Kumar Sen, Wasimul Bari

**Affiliations:** Department of Statistics, University of Dhaka, Dhaka, 1000 Bangladesh

**Keywords:** Quantile, Laplace Survival, Infant Mortality, MICS, Bangladesh

## Abstract

**Background:**

Several studies have identified factors influencing infant mortality, but, to the best of knowledge, no studies assessed the factors considering unequal effects on different survival times of infant mortality in Bangladesh. In this study, it was examined how a set of covariates behaves on different quantile survival times related with the infant mortality.

**Methods:**

Data obtained from Bangladesh multiple indicator cluster survey (BMICS), 2019 have been used for purpose of the study. A total of 9,183 reproductive women were included in the study who gave their most recent live births within two years preceding the survey. Kaplan–Meier product limit approach has been applied to find the survival probabilities for the infant mortality, and the log-rank test has also been used to observe the unadjusted association between infant mortality and selected covariates. To examine the unequal effects of the covariates on different quantile survival time of infant mortality, the Laplace survival regression model has been fitted. The results obtained from this model have also been compared with the results obtained from the classical accelerated failure time (AFT) and Cox proportional hazard (Cox PH) models.

**Results:**

The infant mortality in Bangladesh is still high which is around 28 per 1000 live births. In all the selected survival regression models, the directions of regression coefficients were similar, but the heterogenous effects of covariates on survival time were observed in quantile survival model. Several correlates such as maternal age, education, gender of index child, previous birth interval, skilled antenatal care provider, immediate breastfeeding etc. were identified as potential factors having higher impact on initial survival times.

**Conclusion:**

Infant mortality was significantly influenced by the factors more in the beginning of the infant's life period than at later stages, suggesting that receiving proper care at an early age will raise the likelihood of survival. Policy-making interventions are required to reduce the infant deaths, and the study findings may assist policy makers to revise the programs so that the sustainable development goal 3.2 can be achieved in Bangladesh.

## Background

Though a substantial global improvement has been achieved in child survival and health over the last 35 years [1], sustainable development goal (SDG) 3.2 is yet to reach. The universal rate of under 5 child mortality has declined by 59%, from 93 deaths per 1,000 live births in 1990 to 38 in 2019 [2]. In Bangladesh, the rate of under 5 child mortality has been decreased by 66%, from 134 deaths per 1,000 live births in 1993–94 to 45 in 2017–18 [3]. However, the decline in infant mortality (88/1,000 in 1993 to 38/1,000 in 2017) is slower than under 5 mortality rate [3, 4]. For the last ten years (2007–2017) declining rate in the infant mortality is much lower which is only 27% [3]. The deaths of infants have a significant contribution to under 5 child mortality in Bangladesh, for example, out of 45 under-five child deaths per 1000 live births, 38 were infants [3]. However, the target is to reduce under 5 child mortality to as low as 25 per 1000 live births to achieve sustainable development goal (SDG) 3.2 by 2030 [5]. To achieve this goal, Bangladesh has to further reduce the under 5 child mortality rate [6]. Since infant deaths have a major influence on under-5 mortality, reducing the infant mortality rate will contribute significantly to achieving this SDG. Moreover, studying infant mortality is an important public health issue to improve the maternal and child health in Bangladesh [6].

Despite significant improvements in decelerating child mortality in recent decades, rate of infant and child mortality still remain high in many developing countries [7]. In developing countries, like Bangladesh, progress has been made in the child healthcare system. However, the socioeconomic disparity still exists which is a resistant factor of child mortality [4, 8]. To enhance health services including child health, the government has taken many initiatives. In recent times, services of the public health sectors are almost free of cost. However, poor people have limited access compared to the better offs due to lack of relevant knowledge. In addition, they also have social and cultural barriers to access proper healthcare services [8, 9]. To improve socio-economic and nutritional status, several programs are run by the government and non-government organizations.

Recent studies showed that there exists an inverse relationship between socioeconomic factors and infant mortality [10–13]. Mother’s education is an important factor and has a positive impact on infant mortality [4, 14]. It ensures primary healthcare services such as immunization, growth monitoring, and family planning. Moreover, newborns' early initiation of breastfeeding practice, complementary feeding, and other essential child healthcare practices were influenced by the mother’s education [4, 15, 16]. Various socio-economic and demographic factors were responsible for infant mortality. Household wealth status and maternal and child healthcare services have a direct impact on infant mortality [10, 17].

Several studies were conducted in developing countries to examine the factors affecting infant mortality. Studies found that area of residence [13, 18], wealth index [6, 19], gender of the child [10], longer birth interval between two consecutive births [6, 13, 19, 20], exposure to media [6, 10], antenatal care [4, 19, 21] received from skilled personnel (doctor, nurse/midwife, paramedic, family welfare visitor or community skilled birth attendant) [22, 23], protected against tetanus [13, 22] and immediate breastfeeding practice [15, 24] were significant contributors to reduce infant and child mortality, where logistic regression [10, 13] or different types of survival regression models [4, 6, 25] were used. These studies did not take into account the heterogeneous effects of the covariates on different survival times [26]. Note that classical survival regression is constructed to examine how a set of covariates influence the location parameter of the transformed survival time and but, in practice, it is observed that influence of covariates is higher either on the earlier or later survival times and subsequently influence diminishes as survival time moves to the right or to the left [27]. To accommodate this pattern of influence of covariates a quantile-based Laplace survival regression model is used [28]. To find out potential correlates of the infant mortality, survival data extracted from Bangladesh Multiple Indicator Cluster Survey (MICS), 2019 have been analyzed using quantile survival regression model.

## Methods

### Sample design

The study used the nationally representative dataset extracted from Bangladesh Multiple Indicator Cluster Survey (MICS), 2019 [22]. This survey used the sampling frame constructed by Bangladesh Bureau of Statistics (BBS) in the 2011 for Bangladesh Census of Population and Housing. Using this frame, whole Bangladesh was divided into several strata based on rural–urban areas of each district within each division which consist of enumeration areas (EA) or clusters. A two-stage stratified cluster sampling was used to collect the sample of households in the study. In the first stage, a total of 3220 enumeration areas were selected using probability proportional to size (PPS), and systematic sampling were used to select 20 households from each EAs in the second stage. The Bangladesh MICS, 2019 survey was administered to a total of 64,400 households, and from those, 64,378 women aged 15–49 were successfully interviewed. Detailed sample design was available at the Bangladesh MICS, 2019 report [22].

### Study participants

In the study, a total of 9,183 reproductive women were included in the study who gave their most recent live births within two years preceding the survey. Each woman provides detailed information on her livebirths, including the gender of the child, date of birth, survival status, the current age of the alive child on the date of interview, age at death of each live birth etc.

### Outcome measure

Survival analysis has been conducted in the present study to analyze the infant mortality in Bangladesh. Therefore, the event of interest was whether a child died before celebrating his/her first birthday. The event took value 1 if the child died, otherwise took value 0. Survival time was the age at death (in days) for the event taking value 1, otherwise it was his/her current age (in days).

### Covariates

To assess potential risk factors of infant mortality, socio-economic, demographic, and health-related variables from MICS 2019 data were considered in this analysis based on some previous studies [4, 6, 8, 10, 13, 14, 19, 25]. Maternal age at birth (less than 20 years, 20–34 years, above 34 years), gender of the child (male, female), previous birth interval (1st birth, less than 2 years, 2 years or more) were considered as demographic factors. Moreover, the mother’s level of education (no education, primary, secondary, higher), household wealth index (poor, middle, rich), and exposure to media (yes, no) were taken as socio-economic covariates. In addition, health-related factors such as skilled antenatal care (ANC) provider (yes, no), protection against tetanus (unprotected, protected), and immediate initiation of breastfeeding of the newborns (yes, no) were also selected in the study.

### Statistical analysis

To examine how a covariate influence the survival time, survival probabilities at different time points where events occurred were computed for different categories using product limit approach [29] with log-rank test [30]. In classical survival regression model such as accelerated failure time (AFT) and Cox proportional hazard (Cox PH) models [30], the effects of covariates are assessed on location parameter of probability distribution function of survival time under AFT model and on hazard function under Cox PH model. But it may happen in practice that predictors may have greater effects at an initial period of survival, and weaker effects or even no effect afterward, or vice versa [26, 27]. Moreover, the outcome variable, survival time, is typically skewed; that is, there exists non-normality and long tails, and it is difficult to address these issues in classical survival models. But the quantile-based survival models provide more robust estimation than traditional ones [26]. Laplace survival regression model has widely been used to measure such effects of covariates on different quantiles of survival time [31].

Let ${T}_{i}, i=\mathrm{1,2},\dots ,n,$ be the time to occur an event and ${x}_{i}$ be the k-dimensional vector of observed covariates. In survival setup, ${Y}_{i}=\mathrm{min}\left({T}_{i}, {C}_{i}\right)$ is observed, where ${C}_{i}$ is the censoring time random variable and parameters of the density function of censoring random variable are not of interest. Hence, the censoring indicator, ${\delta }_{i}$, is defined as ${\delta }_{i}=I({T}_{i}\le {C}_{i})$, where $I(\cdot )$ is the indicator function. Laplace quantile survival regression model ca be defined as [28, 32, 33]

1 $${Y}_{i}={x}_{i}^{^{\prime}}{\beta }_{p}+{\varepsilon }_{i},$$

where ${\beta }_{p}$ is the vector of regression coefficients; $p\in (\mathrm{0,1})$, and ${\varepsilon }_{i}$ an independent and identically distributed residuals with $\mathrm{Pr}\left[{\varepsilon }_{i}\le 0 |{X}_{i}\right]=p.$ For any $p\in (\mathrm{0,1})$, the p-quantile of the conditional distribution of ${Y}_{i}$ given ${x}_{i}$ is ${x}_{i}^{^{\prime}}{\beta }_{p}$, i.e., $\mathrm{Pr}\left[{Y}_{i}\le {x}_{i}^{^{\prime}}{\beta }_{p}|{x}_{i}\right]=p.$

As this conditional quantile is equivariant to the non-decreasing transformation of ${Y}_{i}$, it is desirable to use a suitable transformation, say $g\left(\cdot \right)$ so that conditional quantile can be modelled as linear predictor. A popular choice for the transformation is $g\left({Y}_{i}\right)=\mathrm{ln}({Y}_{i})$.

For a given set of covariates (${x}_{i}$), the response, ${Y}_{i}$ follows an asymmetric Laplace distribution with probability density function

$$f\left({y}_{i}|{x}_{i}\right)=\mathrm{exp}\left[\left\{I\left({y}_{i}\le {x}_{i}^{^{\prime}}{\beta }_{p}\right)-p\right\}\frac{{y}_{i}-{x}_{i}^{^{\prime}}{\beta }_{p}}{{\sigma }_{p}}\right]\frac{p\left(1-p\right)}{{\sigma }_{p}},$$

where, ${\beta }_{p} \in \left(-\infty ,\infty \right) and {\sigma }_{p}>0$ are the parameters and the log likelihood function [28] is given as

$${l}_{n}\left\{{\beta }_{p}, {\sigma }_{p}|{y}_{i}, {x}_{i}, {\delta }_{i}\right\}=\sum_{i=i}^{n}\left[{\delta }_{i}\mathrm{log}f\left({y}_{i}|{x}_{i}\right)+\left(1-{\delta }_{i}\right)\mathrm{log}\left\{1-F\left({y}_{i}|{x}_{i}\right)\right\}\right],$$

where, $F(\cdot )$ be the cumulative distribution function. The maximum likelihood estimation technique was used to estimate the parameters of Laplace survival regression model given in Eq. (1). The Weibull AFT and Cox PH models were also considered for the purpose of comparison with quantile survival regression model [30], where 1^st^, 2^nd^ and 3^rd^ quartile coefficients of the covariates were estimated to identify the heterogeneous effects on survival times. Furthermore, to observe the changes of the effects of significant covariates obtained on different quantiles [$p\in (\mathrm{0,1})$], the estimates were also presented graphically. Note that the AFT model can only provide the effects of covariates on location parameters and heterogeneity effects cannot be explored through this model. On the other hand, Cox PH model is developed using the hazard function assuming that constant hazard ratio remains over time [33].

The R package “survival” was used to examine the differences in survival curves for different categories of a covariate and Stata command “laplace” [32] was used to draw inference from Laplace quantile survival regression model.

## Results

### Exploratory data analysis

Out of the 9183 women of whom each reported a live birth, a total of 9183 live births were considered. Among these livebirths, 247 experienced deaths before celebrating their first birthday. It was found that the survival probability at maximum time point was 0.972 which implies that the infant mortality rate was 28 per thousand live births. The percentage of background characteristics of the respondents and the distribution of survival probabilities of infants at different levels of selected covariates obtained from product limit approach along with the *p*-values were reported in Table 1.

**Table 1:** Descriptive statistics and relative infant survival probabilities for the selected covariates, MICS 2019

Covariates		Percentage Distribution	Survival Probability (at most of the time points)	*p*-value
**Maternal Age**	Less than 20 years	20.79	20 to 34 years > Less than 20 years > Above 34 years	0.030
	20 to 35 years	71.98		
	35 + years	7.24		
**Mother’s Education**	No Education	9.17	Higher > Secondary > Primary > No Education	0.008
	Primary	23.24		
	Secondary	50.02		
	Higher	17.58		
**Area of Residence**	Rural	78.08	Rural > Urban	0.600
	Urban	21.92		
**Gender of Child**	Male	52.08	Female > Male	0.030
	Female	47.92		
**Wealth Index**	Poor	29.83	Rich > Middle > Poor	0.020
	Middle	32.14		
	Rich	38.03		
**Previous Birth Interval**	1^st^ Birth	34.98	2 years or more > 1^st^ Birth > Less than 2 years	0.010
	Less than 2 years	5.22		
	2 years or more	59.80		
**Protected Against Tetanus**	Unprotected	17.38	Protected > Unprotected	0.008
	Protected	82.62		
**Skilled ANC Provider**	No	24.80	Yes > No	0.003
	Yes	75.20		
**Media Exposure**	No	34.73	Yes > No	0.010
	Yes	65.27		
**Immediate Breastfeeding**	No	53.43	Yes > No	< 0.001
	Yes	46.57		

Maternal age at birth, mother's education, gender of the child, wealth index, previous birth interval, protection against tetanus, skilled antenatal care provider, media exposure, and immediate initiation of breastfeeding practice were found to have a significant association with infant mortality. It was observed from Table 1 that most of the mothers (71.98%) belonged to the maternal age group of 20 to 34 years, and this category had the lowest infant mortality rate compared to the other two groups (< 20 years and > 34 years). Less than one-tenth of the selected mothers were uneducated; half of the mothers had completed secondary education, and less than one-fifth of the mothers were highly educated. The survival rate was found to be higher among highly educated mothers compared to mothers with lower education levels. Mortality was higher for male children than females. The infant survival rate increases as economic status of household increases. The mortality rate was comparatively greater for the mothers having a shorter birth interval (< 2 years) between two successive live births. Most of the mothers (82.62%) were immunized against tetanus and children born from those mothers were less likely to experience the death before one year of age. Around three-fourths (75.20%) of the mothers received antenatal care from skilled personnel. Infants of the mother receiving antenatal care from unskilled provider died more than their counterparts. Moreover, mothers exposed to the media had more survival rates for their children at most of the time points than mothers not exposed to the media. More than half of the infants (53.43%) were deprived from early initiation of breastfeeding. It was observed that children who were breastfed immediately after birth were less likely to experience infant mortality.

### Laplace survival regression model

**Fig. 1:**
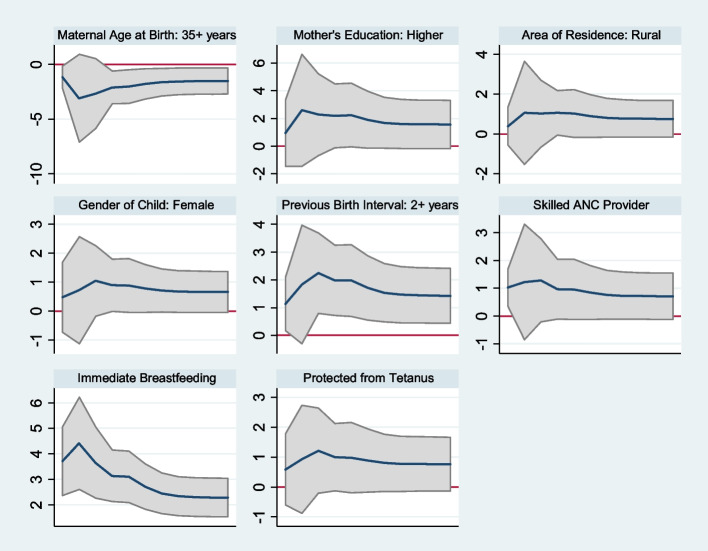
Survival regression coefficients obtained on different quantiles for infant mortality [Y-axis: adjusted effects; X-axis: quantiles (0,1)]

Survival regression coefficients along with robust standard error and *p*-values obtained on the first, second, and third quartiles were reported in Table 2. In Fig. 1, estimates of Laplace survival regression for different quantiles have been plotted along with their 95% confidence intervals for potential covariates to illustrate how the effect of a covariate changes with the quantile values.

**Table 2:** Survival regression coefficients with standard errors in parentheses obtained on the first, second, and third quartiles; as well as from Cox PH and AFT models

Covariates	Quartiles			Weibull AFT	Cox PH
	q = 0.25	q = 0.50	q = 0.75		
**Maternal Age**					
Less than 20 years	0.744 (0.697)	0.633 (0.625)	0.565 (0.565)	0.794 (0.720)	-0.210 (0.189)
20 to 35 years	-	-	-	-	-
35 + years	-2.025** (0.783)	-1.795** (0.706)	-1.630** (0.641)	-2.125** (0.834)	0.563*** (0.216)
**Mother’s Education**					
No Education	-	-	-	-	-
Primary	-0.152 (0.835)	-0.174 (0.756)	-0.173 (0.687)	-0.090 (0.853)	0.016 (0.224)
Secondary	0.306 (0.849)	0.228 (0.767)	0.192 (0.696)	0.393 (0.854)	-0.107 (0.225)
Higher	2.240* (1.175)	1.894* (1.048)	1.685* (0.942)	2.451** (1.160)	-0.647** (0.302)
**Area of Residence**					
Rural	1.021* (0.614)	0.897 (0.550)	0.811 (0.497)	1.064 (0.656)	-0.281 (0.172)
Urban	-	-	-	-	-
**Gender of Child**					
Male	-	-	-	-	-
Female	0.885* (0.473)	0.782* (0.423)	0.711* (0.381)	0.927* (0.498)	-0.243* (0.130)
**Wealth Index**					
Poor	-0.453 (0.568)	-0.412 (0.509)	-0.379 (0.460)	-0.470 (0.627)	0.121 (0.165)
Middle	-	-	-	-	-
Rich	0.686 (0.656)	0.601 (0.584)	0.542 (0.525)	0.722 (0.689)	-0.192 (0.181)
**Previous Birth Interval**					
1^st^ Birth	-	-	-	-	-
Less than 2 years	-0.441 (0.968)	-0.429 (0.871)	-0.408 (0.793)	-0.435 (0.990)	0.121 (0.260)
2 years or more	1.975*** (0.656)	1.710*** (0.589)	1.537*** (0.532)	2.097*** (0.659)	-0.552*** (0.170)
**Protected Against Tetanus**					
Unprotected	-	-	-	-	-
Protected	0.981 (0.599)	0.883 (0.539)	0.809* (0.488)	1.027* (0.597)	-0.266* (0.156)
**Skilled ANC Provider**					
No	-	-	-	-	-
Yes	0.955* (0.554)	0.844* (0.497)	0.764* (0.449)	1.010* (0.574)	-0.270* (0.150)
**Media Exposure**					
No	-	-	-	-	-
Yes	0.678 (0.499)	0.587 (0.447)	0.536 (0.403)	0.677 (0.559)	-0.174 (0.147)
**Immediate Breastfeeding**					
No	-	-	-	-	-
Yes	3.105*** (0.515)	2.719*** (0.455)	2.456*** (0.407)	3.285*** (0.578)	-0.862*** (0.142)

^*^p-value < 0.10, **p-value < 0.05, ***p-value < 0.01

The findings from this study revealed that maternal age was a significant determinant of infant mortality. The survival time at each quartile (${q}_{0.25}, {q}_{0.50} \mathrm{and} {q}_{0.75}$) for the maternal age group 35 + was significantly lower than a group of mothers of age 20–35 years. In the first quartile, the estimated value of the regression coefficient is $-2.025$, which implies children from mothers with 35 + age have 87% lower 25th quantile survival times $[(1-exp(-2.025))\times 100\%]$ compared to the children of 20–35 age group mothers; whereas, the infants of older age mothers have 83% and 80% lower median and third quartile survival times, respectively. Figure 1 depicted that the effects of the older age group (35 +) was low at very initial quantile then effect increases upto quantile 0.025; and it decreases afterward.

Regression coefficients from all three quartiles showed that children from highly educated mothers survived more compared to uneducated. Infants have 839% more first quartile survival for highly educated mothers than the mothers having no education, whereas the median and 0.75th quantile survival times were 562% and 439% greater, respectively. The child from rural area had a 177% greater 0.25th quantile survival time than that from the urban area, but no significant differences were found between these groups at the median and third quartile survival times. Female child had 142%, 118%, and 103% higher 25th, 50th, and 75th percentile survival times, respectively, compared to the male child. Previous birth interval for the index child was a significant predictor of infant mortality. A child born after two years of his/her previous sibling had 453% higher median survival time than a child who was the first child of the parents; and the survival time were 621% and 365% higher at first and third quartile, respectively.

Women protected against tetanus have a 125% higher third quartile infant survival time than their counterparts. However, there was no significant differences at 0.25^th^ quantile and median survival times for protected and unprotected groups. Antenatal care provided by skilled personnel had a significant contribution to decelerate infant mortality. Women receiving skilled antenatal care had 133% higher median infant survival time, whereas the 25th and 75th percentile survival time were 160% and 114% higher, respectively.

Immediate breastfeeding practice of newborns significantly contributed to the infant mortality reduction. The first, second, and third log quartile survival time were 3.105, 2.719, and 2.456 unit higher, respectively, for the infants who had immediate breastfeeding practice compared to the newborns who did not have the opportunity. The wealth index of households and media exposure of women had no significant contribution to infant mortality.

It is apparent from Fig. 1 that the adjusted effect of covariates on infant mortality is generally high on the lower quantiles than upper quantile survival times. This implies that the significant predictors influence survival of infants more during the first days of life.

### AFT and Cox PH regression models

The Weibull AFT and Cox PH regression models were also considered to compare the results with those obtained from Laplace survival regression model. Results computed from AFT and Cox PH models were also presented in Table 2. Maternal age, mother’s education, gender of child, previous birth interval, protected against tetanus, skilled ANC provider and immediate breastfeeding were found to have significant effects on infant mortality in both Weibull AFT and Cox PH models. Babies born from mothers who gave birth at 35 years or later [AFT: Coef. -2.125, *p* < 0.05; Cox: Coef. 0.563, *p* < 0.01] had lower survival time or higher hazard rate compared to babies born from mothers who gave birth before reaching 20 years. Again, babies born from highly educated mothers [AFT: Coef. 2.451, *p* < 0.05; Cox: Coef. -0.647, *p* < 0.05], female babies [AFT: Coef. 0.927, *p* < 0.10; Cox: Coef. -0.243, *p* < 0.10], babies born with interval of 2 or more years of previous birth [AFT: Coef. 2.097, *p* < 0.01; Cox: Coef. -0.552, *p* < 0.01], protected babies against tetanus [AFT: Coef. 1.027, *p* < 0.10; Cox: Coef. -0.266, *p* < 0.10], babies born from mothers who received skilled ANC during pregnancy [AFT: Coef. 1.010, *p* < 0.10; Cox: Coef. -0.270, *p* < 0.10] and babies who took breastfeeding early within 1 h of birth had higher survival time or lower hazard rate.

## Discussion

This study aims to investigate the effects of the determinants on infant mortality using a large-scale nationally representative Bangladesh Multiple Indicator Cluster Survey, 2019 data. The study shows that the infant mortality rate for two years period in 2019 was about 28 per 1000 live births in Bangladesh which underestimates the rate given in the MICS report. This difference may happen due to the consideration of most recent live births born within two years prior to the survey.

To examine the unadjusted association of the selected covariates with infant mortality, the log-rank test was used and the results revealed that all the selected covariates except area of residence were significantly associated with infant mortality at 0.05 level of significance. The Laplace survival regression model was also used to assess the adjusted effects of the socio-economic and demographic factors on survival time at different percentiles such as 25^th^, 50^th^, 75^th^ etc.

The classical models such as Weibull AFT and Cox PH models were also used in the study for comparing with results obtained from Laplace survival model. The results extracted from the models were not directly comparable because Laplace model shows the heterogeneous effects of covariates at different quantiles, whereas Weibull AFT measures the average effect and Cox PH model illustrates the hazard rate of covariates on infant mortality. But the results can be compared with respect to the direction of effects. The adjusted survival regression results indicated that infant deaths depended on maternal age, mother’s education, gender of child, birth interval, skilled ANC provider and immediate breastfeeding practice and similar direction was observed at all selected models. But area of residence and protected against tetanus had shown significant association with infant mortality at 1^st^ and 3^rd^ quartile, respectively, whereas area of residence was not significant in classical models. The heterogenous effects of all the significant factors were observed on infant mortality, where the highest effect was at initial stage (1^st^ quartile) and later it decreased with quartiles, but the classical models were failed to address the effects.

Compared to children born to those mothers in age group 20 to 35 years old, the infant mortality was higher among the children born to older mothers (aged 35 + years). The result was consistent with another study conducted in India [34]. This may be due to high-risk fertility behavior in older women [3, 35]. Pregnancy complications are higher in this group of women than middle aged women. Again, highly educated women had better performance for reducing infant deaths compared to uneducated women. Similar finding was found in Nigeria that women with no education had high hazard rate of infants than those women with secondary or higher education [36]. This is because educated mothers have better knowledge on newborn and child health care, and they are informed regarding utilization of modern healthcare services [37]. Again, uneducated mothers may lead their lives with low standard of living. The study also found that children born to mothers living in rural areas had a lower infant mortality at the first quartile compared with those living in urban areas. The result contradicted the findings of the study conducted in Nigeria [36], Bangladesh [38] and Burkina Faso [39]. This is due to the fact that mothers of the rural area spend more time with newborn compared to those of urban areas. The study showed a little evidence (*p* < 0.10) that male infants are in a higher risk of mortality compared to female infants. This finding was consistent with the results obtained from the studies conducted in Nigeria [36, 40] and India [34]. Previous studies explained that immaturity fetal lung in the first week of life [41] or some biological factors [42–44] may be responsible for high-risk mortality among male child. Gender bias can happen but the study suggests further research to justify the gender difference in infant deaths. Moreover, the risk of infant mortality was lower among mothers who waited at least two years from the previous birth to give the recent birth than those women who become mothers for the first time. Similar finding was also observed in India [34]. This might be happened due to lack of experience to use maternal and child healthcare services among mothers giving first birth. When mothers who received at least two doses of vaccine against tetanus during the pregnancy their babies were more likely to survive compared to the babies whose mothers were unprotected against tetanus at the third quartile. Note that there was no enough evidence to justify the association between protected against tetanus and infant mortality at early stages. Furthermore, the risk of infant mortality was significantly higher for those mothers who did not receive healthcare from skilled health personnel like qualified doctors, nurses, midwives, paramedics, family welfare visitors, or community skilled attendants compared with mothers who received the skilled healthcare during pregnancy period. The pregnancy period is a crucial period for all pregnant women, and several pregnancy complications occurred at this time which may create severe problems for both mothers and newborns. The skilled health personnel are qualified to handle pregnancy complications, and even they can advise women to visit health facilities if serious complications arise. This study revealed that likelihood of infants’ survival is remarkably high for the children if mothers initiated breastfeeding to their newborns within one hour after birth compared to their counterparts. This finding was supported by another study conducted in Bangladesh where it was argued that immediate breastfeeding reduces hypothermia of infants to a great extent [45], and early mother’s milk contains many anti-infective factors and cells which are protective against infection, diarrhea, pneumonia and birth asphyxia for newborns [15, 46].

There are some limitations in the study. Causal relationship may not be justified using MICS, 2019 data as it was collected from a cross-sectional setup. Another limitation of the study is that the survey collected the information from the respondents regarding infants born preceding two years of the survey which may create recall bias.

## Conclusion

The infant mortality rate in 2019 was around 28 per thousand live births in Bangladesh. This rate was higher among the children born to mother who were older at the time of child birth, uneducated, poor, unprotected against tetanus, unskilled ANC receiver or unexposed to media. Furthermore, the infants having shorter birth interval, male or did not start early breastfeeding within one hour of birth had high mortality rate. The classical models, including Weibull AFT and Cox PH, demonstrated the average effect of covariates on survival time, however the quantile survival regression model presented the diverse effects of the covariates on different quantile survival times in the study. Results from the quantile model showed that maternal age at 35 + years, higher education, female gender, 2 or more years of birth interval, skilled ANC provider, and immediate breastfeeding had higher significant influence on infant mortality at the initial stage of survival time of the infant, and the influence rates decreased at later survival times, indicating that adequate care at an early age will increase the survival probability. This message should be circulated among all the reproductive women to prevent their livebirths from early childhood mortality through receiving antenatal care from skilled health professionals, practicing immediate breastfeeding, having wider birth intervals etc. Therefore, the policy-making interventions are required to reduce the infant deaths, and the study findings at initial period of survival time will assist the policy makers to update intervention programs to achieve the sustainable development goal 3.2 in Bangladesh.

## Data Availability

This study used data from Bangladesh Multiple Indicator Cluster Survey (MICS) 2019, which are available at https://mics.unicef.org/surveys.

## Abbreviations

BMICS: Bangladesh Multiple Indicator Cluster Survey
BDHS: Bangladesh Demographic and Health Survey
EA: Enumeration Area
BBS: Bangladesh Bureau of Statistics
SDG: Sustainable Development Goal
ANC: Antenatal Care
PPS: Probability Proportional to Size
